# A statistical method for excluding non-variable CpG sites in high-throughput DNA methylation profiling

**DOI:** 10.1186/1471-2105-11-227

**Published:** 2010-05-05

**Authors:** Hailong Meng, Andrew R Joyce, Daniel E Adkins, Priyadarshi Basu, Yankai Jia, Guoya Li, Tapas K Sengupta, Barbara K Zedler, E Lenn Murrelle, Edwin JCG van den Oord

**Affiliations:** 1 Altria Client Services, Research Development & Engineering, 601 E. Jackson Street, Richmond, VA 23219, USA; 2 Venebio Group, LLC, Virginia Bio-Technology Research Park, Richmond, Virginia, USA; 3 Center for Biomarker Research and Personalized Medicine, School of Pharmacy, Virginia Commonwealth University, Richmond, VA 23298, USA; 4 Memorial Sloan-Kettering Cancer Center, 415 East 68th Street, New York, NY 10021, USA; 5 Bon Secours Virginia Health System, 14331 Roderick Ct., Midlothian, VA 23113, USA; 6 American Type Culture Collection, 10801 University Blvd, Manassas, VA 20110, USA

## Abstract

**Background:**

High-throughput DNA methylation arrays are likely to accelerate the pace of methylation biomarker discovery for a wide variety of diseases. A potential problem with a standard set of probes measuring the methylation status of CpG sites across the whole genome is that many sites may not show inter-individual methylation variation among the biosamples for the disease outcome being studied. Inclusion of these so-called "non-variable sites" will increase the risk of false discoveries and reduce statistical power to detect biologically relevant methylation markers.

**Results:**

We propose a method to estimate the proportion of non-variable CpG sites and eliminate those sites from further analyses. Our method is illustrated using data obtained by hybridizing DNA extracted from the peripheral blood mononuclear cells of 311 samples to an array assaying 1505 CpG sites. Results showed that a large proportion of the CpG sites did not show inter-individual variation in methylation.

**Conclusions:**

Our method resulted in a substantial improvement in association signals between methylation sites and outcome variables while controlling the false discovery rate at the same level.

## Background

DNA methylation involves the addition of a methyl group to DNA resulting in altered gene expression. DNA methylation is essential for normal cell functioning and aberrant methylation has been associated with a variety of developmental disorders and human diseases [1-8]. From a translational perspective, methylation markers hold great promise to be used in clinical settings. That is, the inherent stability of the methyl-cytosine bond renders DNA methylation markers potentially superior to the less stable RNA gene expression markers. Furthermore, methylation markers can be measured using biosamples that are easy to collect (e.g., saliva) and even in archived biosamples. As a matter of fact, diagnostic tools using DNA methylation markers are already under development for early cancer screening [9].

Methylation of human DNA is restricted to CpG sites. Historically, methylation studies typically focused on the CpG sites associated with only a few genes that were hypothesized to be relevant to the disease of interest. However, it has recently become technically and economically feasible to measure the methylation status of (tens of) thousands of genes simultaneously using high-quality commercial arrays [10-12]. Furthermore, the number of sites that can be measured continues to increase rapidly with a number of arrays already providing genome-wide coverage [13-17]. These high-throughput methylation profiling arrays create the opportunity to thoroughly search for methylation markers across the whole genome in a wide variety of biomaterial.

An important question that has received relatively little attention in the methylation literature is whether all CpG sites on the array should be used for subsequent analysis. One potential problem with a standard set of probes measuring the methylation status of CpG sites across the genome is that certain probes may not show methylation differences among individuals in the biosamples that are studied. In order to predict disease status, the CpG sites need to represent methylation variable positions [18] for the specific biosamples and individuals being studied. From a statistical point of view, it is important to exclude "non-variable sites," i.e., CpG sites that show no variation among the individuals studied. The reason is that measurements at these sites would largely reflect experimental "noise" caused by sample preparation, image processing, etc., rather than true biologic differences among the individuals. Consequently, any significant association of these non-variable probes with the outcomes of interest (e.g., disease status) is the result of chance and is therefore a false positive finding. False positive findings are undesirable and should be minimized to avoid wasting time and resources on leads that lack biological relevance. Furthermore, methods to control false positives generally become more stringent in the presence of many markers without effect, thereby sacrificing statistical power to detect true, biologically important methylation markers.

In a sense, the issue resembles the situation in association studies where single nucleotide polymorphisms with very low minor allele frequency (i.e., the marker is not polymorphic) are excluded after quality control analyses. It is also reminiscent of expression array analysis where genes with low intensity or variance are often filtered out prior to further analyses [19,20]. Similar to gene expression, the methylation of many genes is biosample-specific [1]. Efforts are being made to catalogue all methylation variable positions in all major biosamples (Human Epigenome Project or HEP; http://www.epigenome.org). However, this information will not be available in the foreseeable future for the vast majority of CpG sites on commercial arrays and in (human) biosamples. The implication is that a method is needed that can be applied in all scenarios.

Although excluding non-variable CpG sites is relevant in all instances, it may be particularly important for peripheral biofluids, such as blood. Peripheral biofluids are often analyzed when it is not feasible to obtain diseased target tissue. Furthermore, methylation markers that can be measured in peripheral biofluids are potentially much better for diagnostic and prognostic purposes because of the relatively simple, non-invasive manner in which the biosamples can be collected. There is a considerable amount of literature showing that methylation markers are not limited to the affected tissue or cell type, but can be detected in peripheral biofluids. A clear example involves loss of imprinting of IGF2, one of the best-studied epimutations, which is found in the colon as well as lymphocytes and where either methylation marker is associated with increased colorectal cancer risk [21]. Two factors may explain why methylation markers can be detected in peripheral biofluids. First, peripheral blood-based studies may be useful in revealing methylation changes predating or resulting from the epigenetic reprogramming events affecting the germ line and early embryogenesis [22-25]. As the epigenetic profile of somatic cells is mitotically inherited, these epigenetic mutations could be found in cells from peripheral blood. Second, blood contains proteins, metabolites, cells that have been modified as they circulate through diseased tissues and cell-free DNA from diseased tissues and cells. As such, traces of the aberrant methylation in diseased target tissue may be present in peripheral biofluids. The problem here, however, is that methylation markers in peripheral biofluids will not uniquely reflect the physiological and pathophysiological state of the relevant disease tissues. This fact could potentially reduce the ability to detect biological variation in methylation status, and further highlights the need for a method to filter non-variable probes prior to conducting disease or phenotype association tests to improve the statistical power to detect biologically meaningful results.

The goal of this study was to propose and apply a method to estimate the proportion of non-variable CpG sites and exclude those sites from further analyses. Our method essentially uses correlations between technical replicates obtained by assaying the same samples twice. We illustrate our method by analyzing methylation profiles generated using DNA extracted from the peripheral blood mononuclear cells (PBMCs) of 311 human subjects.

## Methods

### Probe and sample correlations

The array signal *y*_ijk _for biosample *i *on probe *j *and replicate number *k *can be written as:

where *m*_*j *_is the average signal at probe *j*, *a*_ij _the biosample specific deviation at probe *j*, and *e*_ij _the measurement error (e.g. be caused by factors related to sample preparation, image processing, etc) for biosample *i *on probe *j *for replicate *k*.

We obtained two replicates, *k *= 1..2, to evaluate the magnitude of the methylation signal versus the measurement error. One way to evaluate this involves calculating for a given probe *j *the Pearson (product moment) correlation between the two replicates using the data from all biosamples. The use of this correlation coefficient assumes that the association between the same probe measured twice is linear. This correlation we will label the "probe correlation". If we assume that for probe *j *the measurement errors are uncorrelated across the two replicates, COV(*e*_i1_, *e *_i2_)_*j *_= 0, the covariance between the measured signals equals the variance of the biosample specific deviations at probe *j*: COV(*y*_i1_, *y *_i2_)_*j *_= VAR(*A*)_*j*_. Furthermore, if we assumes for the sake of simplicity that the precision of the measurements is similar for the two replicates, VAR(*e*_i1_) = VAR(*e*_i2_) = VAR(*E*)_*j*_, then the variance of the measured signals equals VAR(*y*_i1_) = VAR(*y*_i2_) = VAR(*A*)_*j *_+ VAR(*E*)_*j*_. Consequently, the correlation for probe *j *across the two replicates becomes:(1)

where VAR(A)_j _and VAR(E)_j _are random variables obtained as a conditional covariance, and not a fixed quantity obtained from applying the variance functional to an unconditional random variable. This probe correlation is an index of the signal-to-error ratio, as it equals the biological variation in methylation signals across biosamples divided by the total variance that includes the error variance as well.

Equation (1) implies that probe correlations can be low for two reasons. First, the measurement error may overwhelm the true methylation signal so that the probe mainly measures error (i.e., VAR(E)_*j *_>> VAR(A)_*j *_Second, the probe correlation may be low because there is little biological variation in methylation status among biosamples (i.e., VAR(A_j_) ≈ 0). To explore the two possibilities, we can examine the sample correlations as well as the correlation between all probe correlations and the corresponding probe variances.

The sample correlation for a given biosample *i *equals the correlation between the two replicates calculated across the data from all probes. Using assumptions similar to those upon which equation (1) is based, the sample correlation for biosample *i *measured on two occasions equals:(2)

where VAR(M)_*i *_is the variance in methylation signals across all probes for biosample *i *and VAR(E)_*i *_is the variance in the measurement error across all probes for biosample *i*. If measurement error is large relative to differences among probes in their methylation status, in addition to observing low probe correlations, we would expect the sample correlations to be low. In contrast, the combination of low probe correlations and high sample correlations suggests little variation in true methylation across biosamples.

A second way to examine whether low probe correlations are caused by large error variances as opposed to low variances in true methylation status uses all probes to calculate the correlations between technical replicate probe correlations and the total probe variances. If the probe correlation is low primarily due to large measurement errors, we would expect a negative correlation between the probe correlations and the total probe variances. This notion stems from the observation that probes with large error variance, VAR(E)_j_, will on average have large total variance because VAR(Y)_j _= VAR(M)_j _+ VAR(E)_j_, but lower probe correlations, as follows from equation (1). On the other hand, if probe correlations are low because of low variances in true methylation status we would expect a positive correlation. This is because probes with larger variation in true methylation signal, VAR(M)_j_, will on average have larger total variance, VAR(Y)_j_, in addition to larger probe correlations according to equation (1).

### Mixture modeling

Although the above analyses enable us to get a general sense of the magnitude of the true methylation status versus the measurement error, it does not provide specific guidelines about which individual probes to include in further analyses. For this purpose, we propose to use all probe correlations as inputs for a mixture model. This model assumed a mixture of normal distributions (i.e. conditional normality of the probe correlations). A complex issue in mixture modeling involves the choice of the number of underlying distributions/classes [26]. For example, a statistical test for comparing models with different number of classes does not exist. In comparing models with different number of classes, the "best" model is therefore often the one with a substantially better fit according to some information criterion. Furthermore, the interpretation of the classes is critical in choosing the number of classes. In this specific application, the main purpose of the mixture modeling is to eliminate probes showing little variation in true methylation status across biosamples. For that purpose a mixture model where one of the classes has a mean probe correlation close to zero pointing to the probes with no biological variation is sufficient. Based on such an estimated mixture model we can then estimate the (posterior) probability of each probe belonging to the class with the zero probe correlation. If that posterior probability is high, that probe can subsequently be eliminated from further (association) analyses.

We used MATLAB^® ^(The MathWorks, Inc., Natick, MA) to estimate mixture models. MATLAB uses the Expectation-Maximization algorithm to estimate the parameters of the mixture model. In the Expectation step, the posterior probability of each probe is calculated using the current model parameters (i.e., the mixing proportions, means, and variances). In the Maximization step, the model parameters are estimated using the current posterior probabilities. The cycle of Expectation and Maximization steps is repeated until convergence is achieved.

### Application to Illumina^® ^GoldenGate^® ^methylation array

#### Subjects, biosamples and methylation assays

DNA was extracted from whole blood samples from 311 middle-aged and older males and females who had participated in the Lung Health Study (LHS) [27,28] and Genetics of Addiction Project (GAP) at the University of Utah. All participants provided written informed consent, and the blood sampling was part of a study protocol approved by the University of Utah Institutional Review Board. Of the 311 subjects, 145 were cigarette smokers with spirometrically defined chronic obstructive pulmonary disease (COPD) [29], and 166 did not have COPD (91 never smokers and 75 smokers).

The GoldenGate^® ^Assay for Methylation (Illumina Inc., San Diego, CA) was used to assess the DNA methylation status of 1505 CpG sites from 807 genes, simultaneously. Prior to methylation profiling, bisulfite conversion of the DNA biosamples was conducted using the EZ DNA Methylation Kit™ (Zymo Research Corp., Orange, CA) in a 96-well format, as per the manufacturer's protocol; 2 μg of genomic DNA was used for bisulfite conversion. Following conversion, 250 ng of DNA was used for the methylation assay. The BeadStudio^® ^Methylation Module (Illumina Inc., San Diego, CA) was used to read fluorescent signals from scanned images collected from the Illumina Beadarray^® ^Reader.

The 311 DNA biosamples were analyzed using five Illumina GoldenGate matrices. Technical replicates were obtained for 126 biosamples by analyzing each on two separate matrices. The methylation status of each CpG site was calculated based on fluorescent intensities corresponding to the methylated allele (Cy5) and the unmethylated allele (Cy3). In order to remove measurement artefacts prior to calculating the methylation status, Cy3 and Cy5 fluorescent intensities were independently corrected for background signal, as well as differential bisulfite conversion levels between biosamples using an ordinary least squares regression model. Following signal correction, the methylation measurement *y *for biosample *i *on probe *j *was calculated as the ratio of fluorescent intensities from the methylated allele (Cy5) to the total fluorescent signal from both the methylated and the unmethylated alleles (Cy3) such that:(3)

Because this quantity is a ratio, *y*_ij _is a continuous number between 0 and 1. Complete technical details for Cy3 and Cy5 corrections and *y*_ij _calculations are provided in Additional file 1: Supplementary Note 1.

#### Association analyses

The outcomes in this analysis were four measures of lung function or decline in lung function measured spirometrically as the forced expiratory volume in 1 second (FEV_1_) [30]. These four measures were derived by fitting mixed models to longitudinal spirometric, smoking history, and demographic data obtained over the subjects' 17-year average participation period in the LHS and GAP. Conceptually, these measures represent different underlying biological processes driving lung function decline. We focused on age-related decline (Age decline), pack-years-related decline (Pack-years decline), and the intensifying effects of smoking, in terms of number of cigarettes per day (CPD), on decline with age (CPD × Age decline) that together accounted for the vast majority of individual differences in lung function decline in these subjects. In addition, we included Baseline lung function measured at subjects' entry into the study as an outcome measure as it has also been shown to vary in magnitude across individuals [31].

Technical details for the outcome variables are provided in Additional file 1: Supplementary Note 2.

To test for association between DNA methylation variables and lung function decline outcome variables, we performed regression analyses with the probes as predictor variables. The *F*-test statistic was used to perform significance tests. Separate analyses were conducted on all probes as well as on only the subset of probes that remained after selection. Two criteria were used to evaluate the performance of our probe selection method. First, we estimated the proportion of markers without effect (*p*_0_) using the estimator proposed by Meinshausen and Rice [32] which performs well in scenarios where *p*_0 _is close to one. Thus, after successful probe selection, we would expect a smaller proportion of markers without effects. Secondly, we studied the distribution of *q*-values [33,34]. The calculated *q*-values are positive false discovery rates (pFDRs) that use the *p*-value of the tests as threshold for declaring significance. More precisely, if the P-values of the *m *tests are denoted *p*_i_, *i *= 1...*m*, we can estimate the pFDR by:

Thus, the pFDR is estimated by dividing the estimated number of false discoveries (i.e. estimated number of tests for which null hypothesis is true × the probability *t *of rejecting a marker without effect) by the total number of significant tests (i.e. total number of P-values smaller than *t*) that includes the false and true positives. To guarantee that the estimated *q *values are increasing in the same order as the *p *values, q values are estimated as:

Successful probe selection would result in more significant results across a range of pre-specified *q*-value thresholds used to declare significance.

## Results

### Probe selection

**Figure 1:**
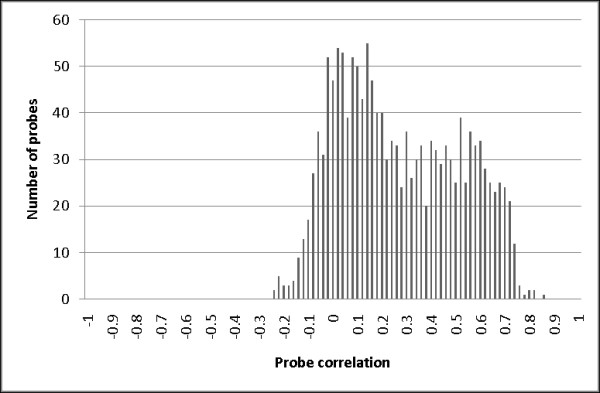
**The distribution of probe correlations**. The distribution of probe-level correlations across technical replicates for each probe is shown. Pearson correlation coefficients were calculated for the 1505 CpG probes using 126 replicate biosamples distributed across five methylation matrices.

**Figure 2:**
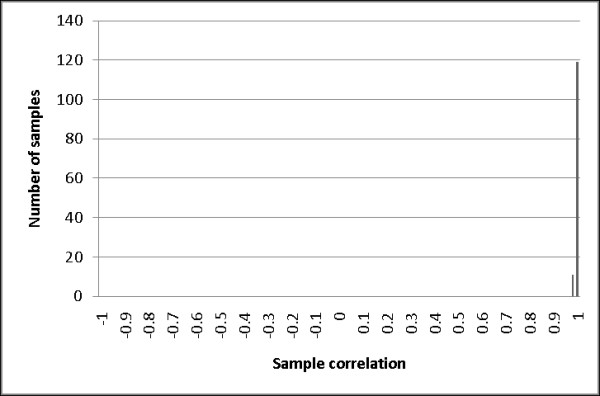
**The distribution of sample correlations**. The distribution of sample correlations across technical replicates for each probe is shown. Pearson correlation coefficients were calculated for the 1505 CpG probes using 126 replicate biosamples distributed across five methylation matrices.

Probe correlations (defined in Equation 1) were calculated using the 126 replicate biosamples and are shown in Figure 1. The mean of probe correlations across the 1505 probes was 0.268 (SD = 0.246). This suggested that, on average, sample differences in methylation status only accounted for only 26.8% of the total variation. Equation (1) indicates two possible reasons for the low probe correlations. First, VAR(E)_j _may be much larger than VAR(A)_j _so that the true methylation signals are overwhelmed by the measurement error. Alternatively, VAR(A)_j_, the methylation difference among biosamples, may be close to zero. To explore whether large error variance versus limited variation in methylation signal caused the small probe correlations, we also calculated the sample correlations (defined in Equation 2) shown in Figure 2. In sharp contrast to the probe correlations, the sample correlations calculated using the 126 replicate biosamples were high, with a mean of 0.995 (SD = 0.0037).

The high sample correlations indicate that the measurement errors are relatively small compared with the methylation variations among probes, because large measurement errors would yield large denominators in Equation 2 and result in low sample correlations. Accordingly, the high sample correlations that we observed suggest that the low probe correlations are not caused by large measurement errors but rather reflect low variation in methylation among the individuals studied. This conclusion was supported when we plotted the correlation between the 1505 probe correlations and the 1505 total probe variances (see Additional file 1: Figure S1). The correlation was 0.436, meaning that probes with high probe correlations also tended to have a relatively larger total variance. This observation also supports the idea that low probe correlations are primarily due to low methylation-related variation among biosamples rather than large measurement errors.

We then attempted to determine which probes should be removed prior to conducting the subsequent statistical analyses. Figure 1 shows that the distribution of 1505 probe correlations is bi-modal. This suggested that probes may fall into two different classes, one with little methylation variation and low probe correlation, and the other with more methylation variation and relatively high probe correlation. Indeed, when we fitted a two-class mixture model the first class had an estimated mean of 0.09 (SD = 0.016) with a mixing proportion of 0.58. These results indicate that nearly 60% of probes had very little variation, highlighting the significance of this probe selection problem and pointing to the probes that should be eliminated prior to association analyses. The second class had an estimated mean probe correlation of 0.51 (SD = 0.019) with a mixing proportion of 0.42. This showed that there were also probes showing variation in methylation that could potentially be disease biomarkers.

**Figure 3:**
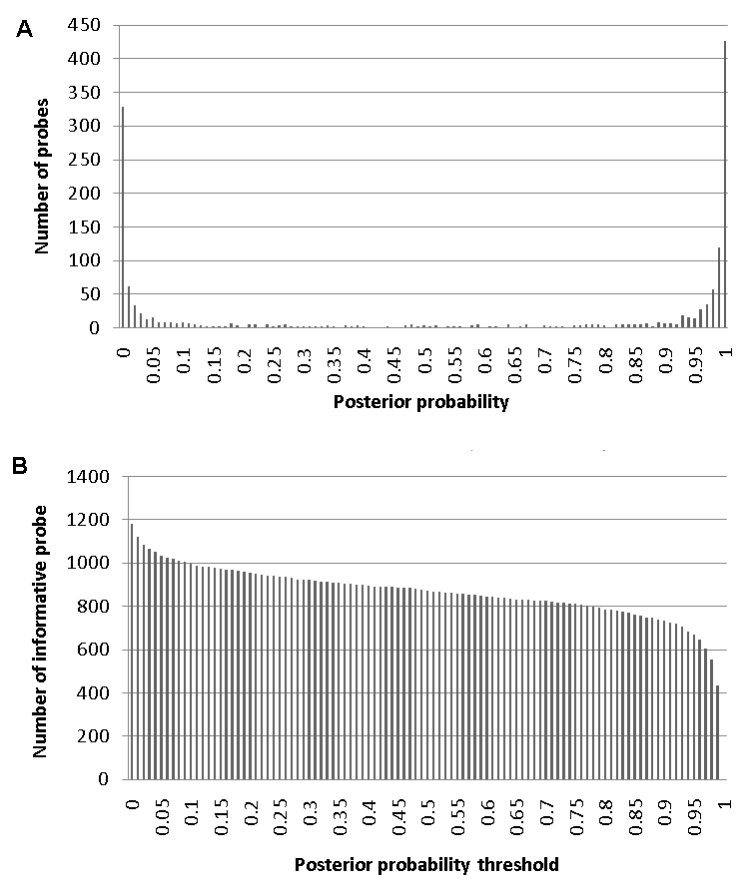
**a - Posterior probability distribution from mixture model**. The posterior probability distribution, indicating the likelihood of a probe belonging to the subset of highly correlated, informative probes. **b - The number of probes selected at different posterior probability thresholds**. The number of probes (y-axis) that will remain at different posterior probability thresholds (x-axis) calculated from the two-class mixture model.

Based on the mixture model, the posterior probabilities of each probe belonging to each class were estimated. The extreme bimodal distribution of the posterior probabilities (Figure 3a) further supported the validity of using a two-class mixture model in this context, and implies that most of the probes can be assigned to one or the other of the classes with reasonably high confidence. Furthermore, the observed bimodality yields the desirable property of cut-off stability where the choice of threshold does not have a major impact on the number of probes selected (Figure 3b). Accordingly, given that probes with higher correlations are more likely to reflect biologically relevant methylation variation, we selected the 634 probes with posterior probability ≥0.5 as members of the class for subsequent analyses.

We found that a class with mean probe correlation of zero was obtained with the two-class model. This makes the two-class model the most parsimonious model for our purpose of eliminating non-variable probes. In addition, Figure 1 shows that the two-class solution has good face validity as the probe correlation distribution was clearly bimodal and Figure 3 shows that the two-class solution gives an excellent separation of the two components where very few probes having a substantial posterior probability of belonging to both classes. For sake of completeness, in Additional file 1 figure S2 we also plotted two fit indices (AIC and BIC) for solutions with 1-6 classes. S2 shows a dramatic improvement in fit going from a one- to two-class solution with very little additional improvement in fit using more than two classes. Such plots too have been proposed as a selection criterion [26] and suggest here that a two-class solution may be the most parsimonious mixture model for the purpose of selecting probes showing no inter-individual variation.

### Association analysis

**Figure 4:**
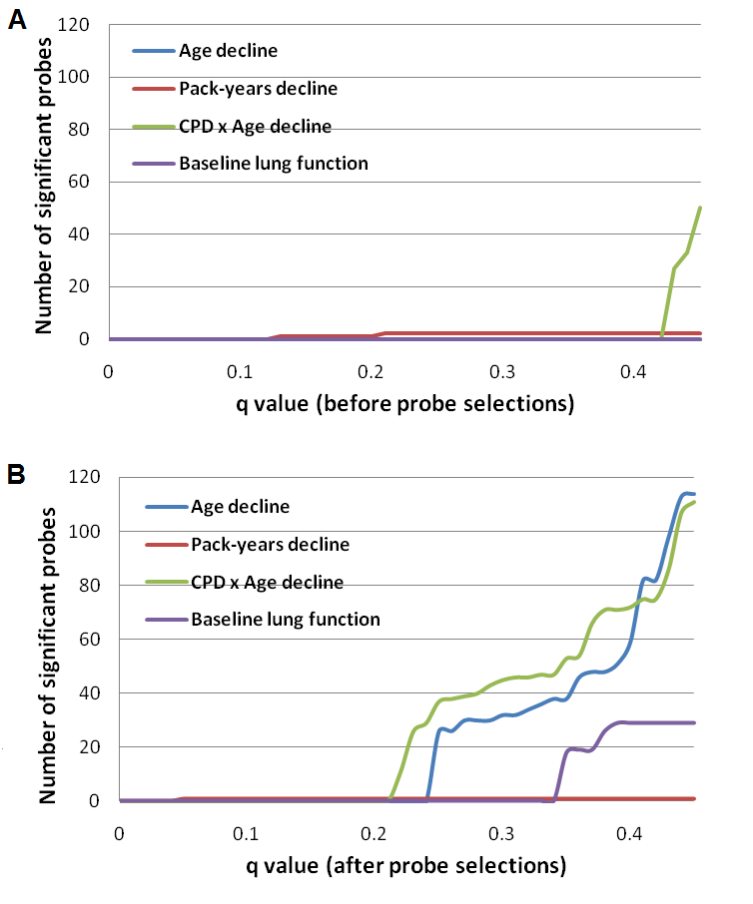
**pFDR analyses**. (A) The number of significant probes detected at different *q*-value thresholds from the regression analyses between DNA methylation changes and four outcome measures of lung function or decline prior to probe selection described herein. (B) The number of significant probes detected at different *q*-value thresholds after probe selection. A greater number of significant probes was identified for a given *q*-value cutoff for Age decline, CPD × Age decline and Baseline lung function outcomes after probe selection.

To evaluate the statistical advantages of the proposed probe selection method, we first estimated the proportion of probes without effect (*p*_0_). Table 1 shows that *p*_0_s after probe selection were consistently lower than *p*_0_s before probe selection. In an absolute sense the improvement in *p*_0_s is not large, but this is because we do not expect *a priori *that a large number of methylation probes are associated with the outcome of interest. Figure 4 displays the distribution of *q-*values before (Figure 4A) and after (Figure 4B) probe selection, providing a better conceptual representation of the practical impact of probe selection. A substantial improvement, as indicated by an increase in the number of identified significant probes, was observed for the outcome variables Age decline, CPD × Age decline and Baseline lung function. At the same pFDR level, the number of significant probes consistently increased. In other words, probe selection seems to improve the statistical power to find probes that are associated with the outcomes while controlling the false discovery rate at the same level.

**Table 1:** *p*_0 _estimates using test results from regression analyses

Outcome	Before probe selection	After probe selection
Age decline	0.9996	0.9781
Pack-years decline	0.9992	0.9986
CPD × Age decline	0.9970	0.9715
Baseline lung function	1.0009	0.9904

CPD, cigarettes per day.

## Discussion

New high-throughput methylation profiling arrays offer the opportunity for systematic searches for methylation markers across a wide variety of biomaterial. The potential problem with a standard set of probes measuring the methylation status of CpG sites across the whole genome is that a proportion of the sites may not show inter-individual methylation variation in the biosamples for the disease outcome being studied. In this article, we propose a method to estimate the proportion of non-variable CpG sites and to exclude those sites from further analyses. Excluding such CpG sites prior to further analyses is important to minimize false-positive findings and to improve statistical power to detect true, biologically relevant methylation markers.

We applied our method to methylation profiles generated using DNA extracted from the PBMCs of 311 subjects. Approximately 60% of the CpG sites did not show inter-individual variation in methylation. The probes on the array used in this study involve a cancer panel that was studied using whole blood collected from patients without cancer. It may therefore very well be that in cancer tissue, more probes would show inter-individual variation. However, arrays with a standard set of probes measuring the methylation status of CpG sites across the whole genome for high-throughput methylation profiling arrays are increasingly used to search for methylation markers across the whole genome in a wide variety of biomaterial. The point we would like to convey is that a (large) proportion of CpGs on such standard arrays may not vary with respect to methylation status. Inclusion of these so-called "non-variable sites" will increase the risk of false discoveries and reduce statistical power to detect biologically relevant methylation markers. Furthermore, we expect the same problem to apply to technologies that sequence the methylated fraction of the genome. That is, part of the methylomes may not show inter-individual variation.

In our analyses, the FDR was used to illustrate our probe selection method. The apparent gain in power we observed should generalize to other methods for controlling false discoveries. For example, when a set or family of *m *tests is performed, we can also control the family-wise error rate (FWE) ensuring that the probability of one or more false discoveries is less than the chosen significance level, *a*. The Bonferroni correction is a simple and commonly used method to control the FWE. The threshold *p*-value (*α*') used to declare significance computed with the Bonferroni correction is *α*' = *a/m*. This simple equation shows that a reduction in the number of probes (*m*) using a probe selection method such as the one described herein results in a much more liberal threshold *p*-value and hence better power to detect true methylation markers. For our follow up association analyses, probes were either included or excluded. This binary choice seemed justified by the bimodal distribution of the posterior probabilities (Figure 3) suggesting that the vast majority of probes could be assigned to one of the two categories with high probability. It is possible that in other scenarios the posterior probabilities may follow a more continuous distribution making the decision of which probes to exclude more arbitrary. In these scenarios an alternative would be to include all probes and then weigh them differentially in the analysis (e.g., by *p*-value weighting) [35] according to their posterior probabilities of showing variability or not. For example, a probe with a high probability of showing no variability would have a weight close to zero and would essentially be excluded from the analysis, while a probe with a high probability of showing variability would get a large weight because any association with the outcome of interest would much more likely be true.

We explored several variables to see if they could predict the variable sites. First, we examined whether probe correlation could be predicted from methylation levels. However, the correlation between methylation levels and probe correlation was 0.205 (Additional file 1: Figure S3). This very modest correlation means that a simple rule excluding all probes that never exceed a certain percentage methylation will not perform as well as the proposed method.

Second, Bock at al. [36] studied 1,705 amplicons (average size 287 bp) from human chromosomes 6, 20 and 22 in three tissues (CD4+ T lymphocytes, CD8+ T lymphocytes and melanocytes) from 10 different biosample donors. These authors considered total variation as a measure of inter-individual differences. One of their main findings was that probes that were more likely to show inter-individual differences in methylation were less likely to be in CpG islands. Although our measure of inter-individual differences is more refined (e.g. the total variance measure used by Bock et al. may be high because of a large error component rather than large inter-individual differences in methylation), we did observe a correlation of .436 (Additional file 1: Figure S1) between our measure and the total variance meaning that the two measures are related. Using our measure of inter-individual differences, we observed that 61.2% of the probes classified as being variable were in CpG islands versus 75.4% of the probes classified as being non-variable. We therefore replicate the findings of Bock et al. Implications of this finding are that, for example, focusing on CpG islands (as some commercial arrays do) could be somewhat limited in terms of explaining variation in diseases.

Third, Christensen et al. [37] used the same array as the one in the present study to compare methylation patterns across 11 tissues from 217 samples. We downloaded their spreadsheet reporting the mean probe methylation levels of all 11 tissues. For each probe we calculated the variance across the 11 tissue means and then correlated the probe correlations from our study with these between-tissue variances. The correlation was 0.29 (see also Additional file 1: Figure S4). Because tissues came from different subjects in the Christensen et al. study, the between-tissue variance is a function of both differences between tissues as well as differences between subjects. However, since the between-tissue variance heterogeneity was much larger than the within-tissue heterogeneity and within-tissue heterogeneity was reduced because we used the mean across all subjects to estimate the tissue specific methylation levels, the between-tissue variance will for an important part capture differences between tissues. Thus, these analyses provide some support for the hypothesis that probes showing more inter-individual variation are more likely to show variation across different tissues.

In this study we used two technical replicates. More technical replicates could be used to improve the probe selection. The mixture modelling could then be performed using interclass correlations (e.g. as estimated using mixed models) as input. How much the probe selection can be improved will depend on factors such as the measurement error and amount of biological variation. Furthermore, rather than doing more replicates for the same number of biosamples, probe selection can also be improved by increasing the number of biosamples for the same number of replicates. Optimization algorithms can in principle be constructed to determine the optimal balance between number of replicates and biosamples for a given budget.

Our approach provides a statistical framework to filter out non-variable probes in any analysis, regardless of the individuals, biosamples, or CpG sites that are studied. The technical replicates are a key component of this approach. Extra methylation arrays and lab work for those replicates may increase the cost of the study. However, since the probe selection method improves the power to detect disease-relevant methylation markers and aims to minimize unnecessary false discoveries, a substantial savings in time and resources can be potentially achieved in the subsequent biomarker verification process. Furthermore, for a given scenario, methylation profiles for technical replicates need to be performed only once. The probes showing no variation could then be catalogued and excluded from future studies that use similar biosamples. However, given the tremendous variability in individuals that are studied (e.g., having different (sub)types of diseases), biosamples that are used, and CpG sites that are interrogated by the different arrays, it is unlikely a comprehensive catalogue can be established in the near future. For many research scenarios, the use of a framework such as proposed here to filter out non-variable probes may be the best alternative.

## Conclusion

In this study, we proposed a statistical method to estimate the proportion of non-variable probes and eliminate those probes from further analyses. Excluding those non-variable probes resulted in a substantial improvement in association signals between probes and outcome variables while controlling the false discovery rate at the same level.

## Potential Conflict of interests

EvdO was a consultant for Altria Client Services. The Center for Biomarker Research and Personalized Medicine was made possible in part by a start-up gift from Altria Client Services Inc. to the VCU School of Pharmacy. ELM, BKZ and ARJ were employees of Altria Client Services at the time of manuscript preparation. The other authors declare no potential conflicts of interest. The authors alone are responsible for the content and writing of the paper.

## Authors' contributions

HM, ARJ, and EvdO developed the method. HM, ARJ, and DEA performed data analysis. PB conducted bisulfite conversion of the biosamples. YJ carried out the DNA methylation array experiments. HM, ARJ and EvdO drafted the manuscript. GL, TKS, BKZ, and ELM participated in method design and edited the manuscript. All authors read and approved the final manuscript.

## Supplementary Material

Additional file 1 **Supplementary Material**. Technical details of the study, including methylation status calculation, measures of lung function and decline, and supplementary figures. Click here for file
