# Supplementary material for: A statistical method for excluding non-variable CpG sites in high-throughput DNA methylation profiling
Source: BMC Bioinformatics. 2010 May 5;11:227. doi: 10.1186/1471-2105-11-227 (PMC2876131; doi:10.1186/1471-2105-11-227)
Supplement: Additional file 1 — Supplementary Material. Technical details of the study, including methylation status calculation, measures of lung function and decline, and supplementary figures. [file 1471-2105-11-227-S1.PDF]

SUPPLEMENTARY MATERIAL

A statistical method for excluding non-variable CpG sites in high-throughput DNA methylation profiling

Meng H., et al.

Supplementary Note 1 – Methylation Status

Prior to calculating the methylation status, fluorescent intensities (Cy3 and Cy5) were normalized to remove measurement artefacts. Illumina® provides two standard normalization methods denoted as the background normalization and average normalization method, respectively. The background normalization method subtracts a background value calculated by averaging the signals of built-in negative controls, whereas the average normalization method averages the signals across multiple arrays. However, in this study we developed a slightly different approach to capitalize on additional characteristics of the DNA methylation array. Specifically we corrected for Cy3 and Cy5 fluorescent intensities independently and also corrected for differential bisulfite conversion levels across samples using an ordinary least squares (OLS) regression model.

To estimate fluorescent signals in the absence of hybridization as a means to assess background signal intensity, principal components analysis (PCA) was performed on the 22 built-in negative controls. Those negative controls are probes that lack a specific target in the genome and are included on the GoldenGate® Assay for Methylation (Illumina Inc., San Diego, CA) for each biosample. Since the independent variables in the OLS regression model are assumed to be independent, we applied PCA to transform the 22 negative control signals into orthogonal principal components. The first 10 principal component (PC) scores (PC_{cy3} and PC_{cy5}) were selected for inclusion in the model. While each of the 10 PC scores is not likely to be required to remove the artefactual background signal, we nonetheless chose to be more inclusive given that PCs that are not predictive will have regression model coefficients (or weights) close to zero and thus have essentially no effect on the final adjusted value. The Cy3 signals were corrected not only by Cy3 background signal but also by Cy5 background signals since the relevance was found between Cy3 signals and Cy5 background signals. In the same way, the Cy5 signals were corrected by both Cy5 and Cy3 background signals. The Cy5/Cy3 ratios of two built-in bisulfite conversion (BC) control probes also were included in the model to correct for any bisulfite conversion differences among biosamples. The resulting regression model was constructed for each methylation probe and each GoldenGate assay matrix to normalize Cy3 and Cy5 signals separately as follows:

$$Cy3 = \beta_0 + \sum_{i=1:10}(\beta_i \times PC_{cy3i}) + \sum_{j=1:10}(\beta_j \times PC_{cy5j}) + \sum_{k=1:2}(\beta_k \times BC_k) + \varepsilon$$

$$Cy5 = \beta_0 + \sum_{i=1:10}(\beta_i \times PC_{cy3i}) + \sum_{j=1:10}(\beta_j \times PC_{cy5j}) + \sum_{k=1:2}(\beta_k \times BC_k) + \varepsilon$$

where Cy is the fluorescent signal (either $Cy3$ or $Cy5$), β_0 is the intercept term, β_i are the coefficients associated with PC_{cy3i}, β_j are the coefficients associated PC_{cy5j}, β_k are the coefficients associated with BC_k, and ε is the residual.

Normalized Cy3 and Cy5 signals were calculated as the sum of the global mean of Cy3 and Cy5 for the CpG site across matrices and their residual in the above regression analysis. Cy3 signals of some probes targeting fully methylated sequences are expected to have negative signals when signals of negative controls were regressed out during the normalization. The same is true for Cy5 signals for some probes targeting fully unmethylated sequences. To avoid potential problems introduced by including negative values, Cy3 and Cy5 were adjusted such that all signals are positive and the smallest value is 0.01.

The methylation level y of each CpG site was calculated as the ratio of adjusted intensities between methylated and unmethylated alleles as follows:

$$y = \frac{Cy5}{Cy5 + Cy3}$$

This quantity was then used in the subsequent probe selection and association testing procedures.

Supplementary Note 2 – Measures of lung function and decline

The outcome variables used in these analyses were derived from random effects in linear mixed models analyzing longitudinal spirometric, smoking history, and demographic data [1]. Specifically, data was modelled for 624 cigarette smokers with chronic obstructive pulmonary disease (COPD) and aged 35–60 at baseline, followed up 7 times over approximately 17 years (1986–2004) in the Lung Health Studies [2,3] and its follow-on Genetics of Addiction Project (GAP); 204 GAP subjects without COPD were also studied (see Supplementary Table 1 for descriptive statistics). The optimal model of the data was selected based on likelihood ratio tests, which were

used to determine the significance of each fixed and random effect parameter as it was added to the model [4]. After the optimal model was identified, the outcome variables were calculated as best linear unbiased predictors (BLUPs) of the random effects. Missing data were handled by multiple imputation using chained equations, with 5 datasets imputed and analyzed [5,6].

Supplementary Table 1. Descriptive statistics of subject characteristics at study initiation*

	Female (N = 303)		Male (N = 525)	
Variables	Mean ± SD	Range	Mean ± SD	Range
Age (y)	44.82 ± 8.08	26 - 60	46.59 ± 7.47	28 - 68
FEV_1 (L)	2.44 ± 0.52	1.18 - 3.93	3.16 ± 0.63	1.02 - 6.09
Height (cm)	164.01 ± 5.88	150 - 180	176.89 ± 6.37	151 - 197
Pack-years	28.41 ± 20.44	0 - 87.5	38.14 ± 23.29	0 - 153
CPD	0.58 ± 0.60	0 - 2.71	0.77 ± 0.67	0 - 4
Never smoked	0.21	0 - 1	0.09	0 - 1
Total missing data, all variables and waves	8.81%		8.73%	

CPD, cigarettes per day. Note: Due to extremely small coefficient sizes, CPD was specified as CPD / 20, thus making the measurement equivalent to packs per day; FEV_1, forced expiratory volume in 1 second; SD, standard deviation.

*Descriptive statistics calculated from non-imputed data at participant's first assessment.

In developing the random effect-based outcome measures, we systematically developed linear mixed models predicting forced expiratory volume in 1 second (FEV_1). Linear mixed models are a generalization of linear regression allowing for the inclusion of random deviations (i.e. random effects) other than those associated with the overall residual term. In matrix notation,

$$y = X\beta + Zu + \varepsilon$$

where **y** is the $n \times 1$ vector of responses, **X** is a $n \times p$ design/covariate matrix for the fixed effect **β**, and **Z** is the $n \times q$ design/covariate matrix for the random effects **u.** The $n \times 1$ vector of residuals **ε,** is assumed to be multivariate normal with mean zero and variance matrix $\sigma_e^2\mathbf{I}_n$.

The fixed portion, **Xβ**, is equivalent to the linear predictor of OLS regression. For the random portion, **Zu** + **ε**, it is assumed that the **u** has variance-covariance matrix **G** and that **u** is orthogonal to **ε** so that

$$\mathrm{Var}\begin{bmatrix}\mathbf{u}\\ \varepsilon\end{bmatrix} = \begin{bmatrix}\mathbf{G} & 0\\ 0 & \sigma_e^2\mathbf{I}_n\end{bmatrix}$$

The random effects **u** are not directly estimated (although, as described below, they may be predicted), but instead are characterized by the elements of **G**, known as the variance components, that are estimated along with the residual variance σ_e^2. Considering **Zu** + **ε** the combined error, we see that **y** is multivariate normal with mean **Xβ** and $n \times n$ variance-covariance matrix

$$V = ZGZ' + \sigma_e^2 I_n$$

The model building process is shown in Supplementary Table 2. The outcome measures used in this analysis were derived from the random effects of the final, best-fitting model:

$$y_{ij} = \beta_0 + \beta_1 x_{1ij} + \beta_2 x_{2ij} + \beta_3 x_{3ij} + \beta_4 x_{4ij} + \beta_5 x_{5ij} + \beta_6 x_{6ij} + \beta_7 x_{7ij} + u_{0i} + u_{1i} + u_{2i} + u_{3i} + e_{ij}$$

where i indexes subjects, j indexes repeated assessments, y is FEV_1, β_0 is the intercept fixed effect, x_1 is age, β_1 is the age fixed effect, x_2 is pack years, β_2 is the pack years fixed effect, x_3 is CPD × age, β_3 is the CPD × age fixed effect, x_4 is height, β_4 is the height fixed effect, x_5 is gender, β_5 is the gender fixed effect, x_6 is gender × age, β_6 is the gender × age fixed effect, x_7 is never-smoked status, β_7 is the never-smoked status fixed effect, u_{0i} is the intercept random effect, u_{1i} is the age random effect, u_{2i} is the pack years random effect, u_{3i} is the CPD × age random effect and e_{ij} is the within-subject residual. Parameter estimates and p-values for the final model (shown in Supplementary Table 2 as Model 15) are shown in Supplementary Table 3.

Supplementary Table 2. Results of FEV_1 linear mixed modeling

Model	Variables	Test statistic*	*df*†		vs. Model	*p*-value
1	Intercept	-		-	-	-
2	Model 1 + Random Intercept	2423.13	1,	41	1	< .001
3	Model 2 + Age	992.28	1,	25	2	< .001
4	Model 3 + Random Age	99.30	1,	159	3	< .001
5	Model 4 + Unstructured RE covariance	122.74	1,	128	4	< .001
6	Model 4 + Age^2	2.48	1,	17	5	NS
7	Model 5 + Height	283.98	1,	110	5	< .001
8	Model 6 + Male	26.38	1,	137	7	< .001
9	Model 7 + Male × Age	15.00	1,	1144	8	< .001
10	Model 8 + Height × Age	3.80	1,	65	9	NS
11	Model 8 + Pack-years	14.56	1,	6	9	< .01
12	Model 10 + Random Pack-years	51.35	1,	7	11	< .001
13	Model 11 + CPD × Age	7.89	1,	7	12	< .05
14	Model 11 + Random CPD × Age	27.96	1,	18	13	< .001
15	Model 12 + Never smoked	104.69	1,	248	14	< .001
16	Model 13 + CPD	1.03	1,	41	15	NS
17	Model 13 + Pack-years × Age	0.46	1,	164	15	NS
18	Model 13 + Never smoked × Age	0.36	1,	19779	15	NS

CPD, cigarettes per day. Note: Due to extremely small coefficient sizes, CPD was specified as CPD / 20, thus making the measurement equivalent to packs per day; FEV_1, forced expiratory volume in 1 second; RE, random effect; NS, not significant.

*This is the multiple imputation version of the likelihood ratio test statistic [7,8]. The test statistic approximates an *F*-distribution under the null hypothesis. See Bollen and Curran [9] for test statistic and degrees of freedom equations.

†Two values are given for the degrees of freedom as the test statistic has an *F*-distribution.

The covariance structure of the four random effects was modeled as unstructured:

$$\begin{bmatrix} u_{0i} \\ u_{1i} \\ u_{2i} \\ u_{3i} \end{bmatrix} \sim N(0,\mathbf{G}) \qquad \text{with } \mathbf{G} = \begin{bmatrix} \sigma^2_{u0} & & & \\ \sigma_{u10} & \sigma^2_{u1} & & \\ \sigma_{u20} & \sigma_{u21} & \sigma^2_{u2} & \\ \sigma_{u30} & \sigma_{u31} & \sigma_{u32} & \sigma^2_{u3} \end{bmatrix}$$

Thus, the random parameters are multivariate normal distributed with means of zero and variance-covariance matrix **G**. The variances of the parameters are on the diagonal and the covariances in the off-diagonal cells of **G**. The residual is assumed to be normally distributed with a mean of zero and variance of σ^2_e.

Because random effects are not directly estimated by the mixed model, they must be predicted in an additional post-estimation step. BLUPs of the random effects **u** were obtained as

$$\tilde{\mathbf{u}} = \tilde{\mathbf{G}}\mathbf{Z}'\tilde{\mathbf{V}}^{-1}(\mathbf{y} - \mathbf{X}\hat{\beta})$$

where $\tilde{\mathbf{G}}$ and $\tilde{\mathbf{V}}$ are **G** and **V** with estimates of the variance components plugged in. The EM algorithm was used for maximum likelihood estimation as described by Pinheiro and Bates [10].

Supplementary Table 3. Parameter estimates and statistical significance of final linear mixed model of FEV_1

	Parameters	SE	*p*-value
Fixed Effects			
Intercept (L)	2.960	0.047	< .001
Age (y)	-0.027	0.002	< .001
Height (cm)	0.031	0.002	< .001
Male Gender	0.542	0.055	< .001
Height × Age	-0.009	0.002	< .001
Pack-years	-0.002	0.001	< .05
CPD × Age	-0.003	0.000	< .01
Never smoked	0.780	0.064	< .001
Random Effects			
SD (Intercept)	0.505	0.031	< .001
SD (Age)	0.021	0.001	< .001
SD (Pack-years)	0.008	0.002	< .001
SD (CPD × Age)	0.007	0.001	< .001

CPD, cigarettes per day. Note: Due to extremely small coefficient sizes, CPD was specified as CPD / 20, thus making the measurement equivalent to packs per day; FEV_1, forced expiratory volume in 1 second; SD, standard deviation; SE, standard error.

Supplementary Note 3 – Figures S1 to S4

The relationship between probe correlations and total probe variances is shown in Figure S1.

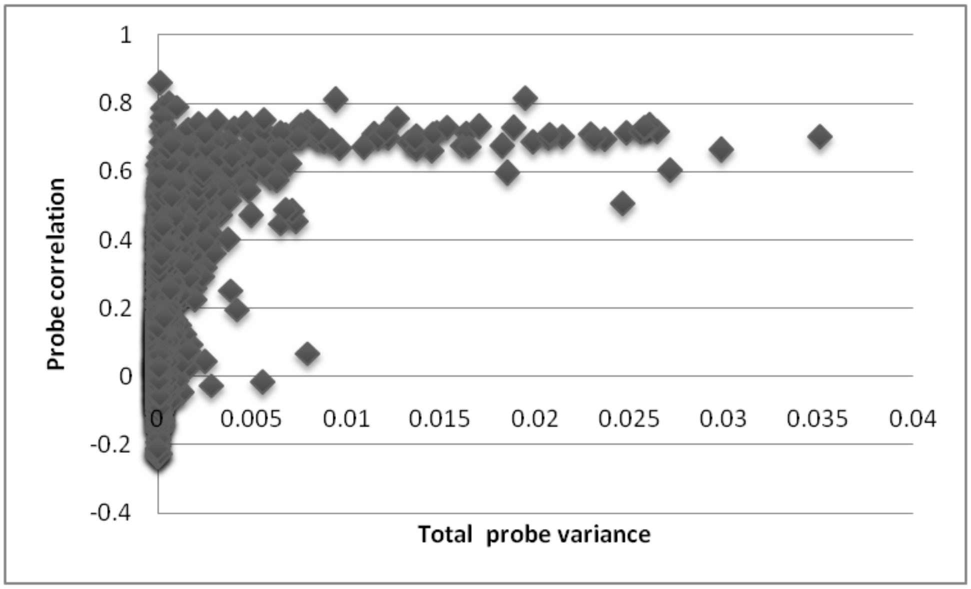

Figure S1. Plot of probe correlation versus total variance

Figure S1 shows that a relatively high total probe variance tends to correspond to a high probe correlation across technical replicates.

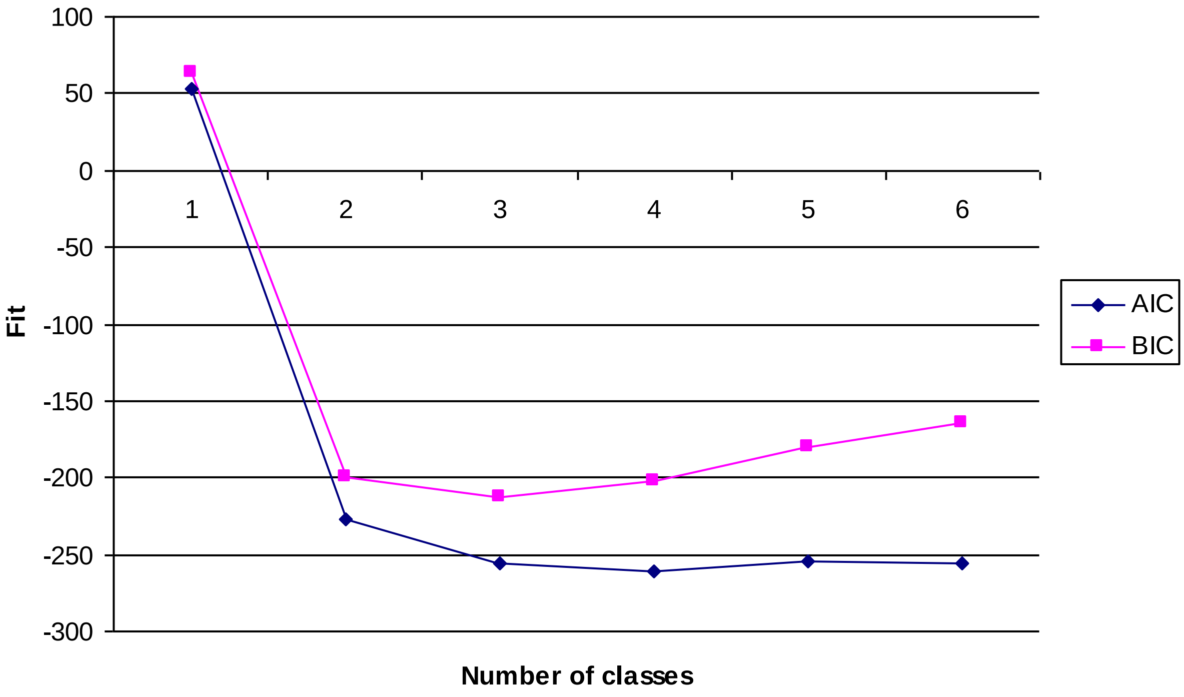

Figure S2. Fit indices (AIC and BIC) of mixture models with 1-6 classes using probe correlations as input.

Figure S2 shows a “scree” plot using the fit indices for solutions with 1-6 classes. It shows a “jump” in fit going from 1 to 2 classes and that relatively little is gained using more than two classes. This “scree” tests suggest a two-class solution may be to most parsimonious mixture model for the purpose of selecting probes showing no inter-individual variation. .

Figure S3 shows the relation between the probe intensities (beta value) and probe correlations.

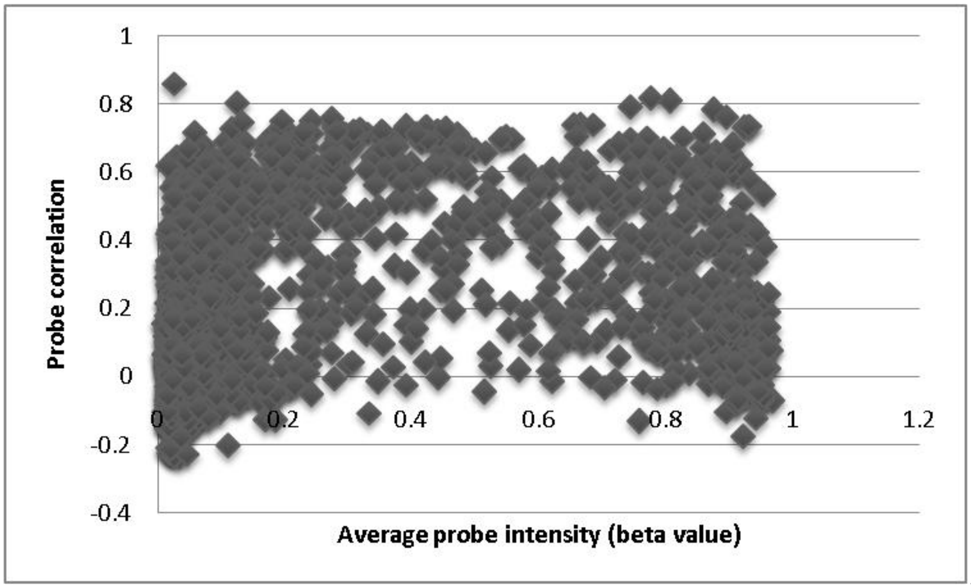

Figure S3 Plot of mean of beta versus probe correlations

Results suggest that the probe intensity is not a good predictor of variability in methylation status.

Finally, Christensen et al. [11] used the same array as the one in the present study to compare methylation patterns across a variety of tissues. We downloaded their data from 217 samples and calculated whether the variable probes in our analysis were more likely to show variation in methylation status across the 11 tissues in the Christensen et al. study. Figure S4 plots the results and the correlation was 0.29.

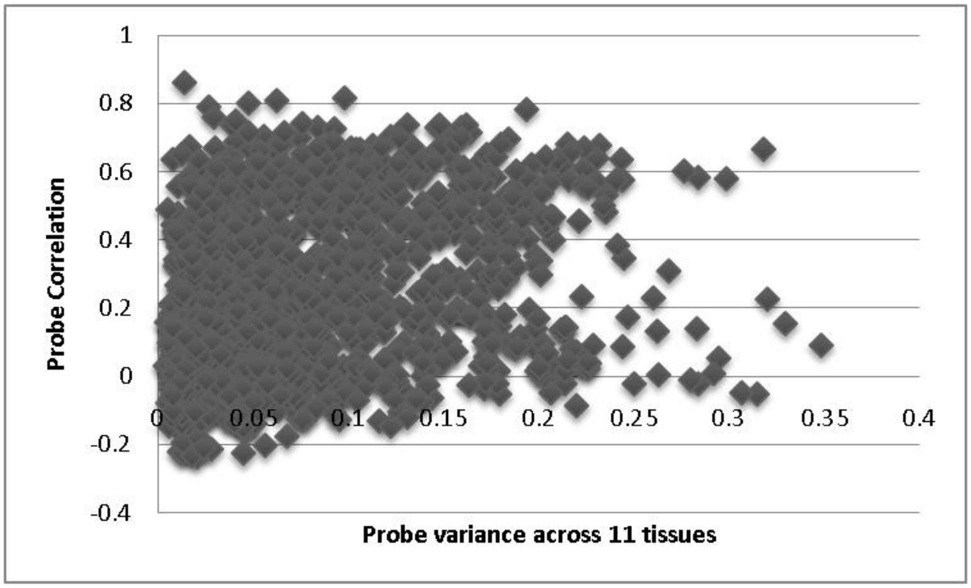

Figure S4. The variance across 11 tissues (data from Christensen et al [11]) and probe correlations calculated from our data.

Supplementary References

1. Goldstein H: *Multilevel statistical models.* New York: Wiley; 1995.
2. Connett JE, Kusek JW, Bailey WC, O'Hara P, Wu M: **Design of the Lung Health Study: a randomized clinical trial of early intervention for chronic obstructive pulmonary disease.** *Control Clin Trials* 1993, **14:**3S-19S.
3. Anthonisen NR, Connett JE, Kiley JP, Altose MD, Bailey WC, Buist AS, Conway WA, Enright PL, Kanner RE, O'Hara P: **Effects of smoking intervention and the use of an inhaled anticholinergic bronchodilator on the rate of decline of FEV1: the Lung Health Study.** *JAMA* 1994, **272:**1497-1505.
4. Willet JB, Singer JD, Martin NC: **The design and analysis of longitudinal studies of development and psychopathology in context: Statistical models and methodological recommendations.** *Dev Psychopathol* 1998, **10:**395-426.
5. Van Buuren S, Brand JPL, Groothuis-Oudshoorn K, Rubin DB: **Fully conditional specification in multivariate imputation.** *J Stat Comput Sim* 2006, **76:**1049-1064.
6. Royston P: **Multiple imputation of missing values: update.** *S J* 2005, **5:**527-536.
7. Li K, Raghunathan T, Rubin D: **Large sample significance levels from multiply-imputed data using moment-based statistics and an F reference distribution.** *JASA* 1991, **86:**1065-1073.
8. Allison P: *Missing data.* Thousand Oaks, CA: Sage Publications, Inc.; 2002.
9. Bollen KA, Curran PJ: *Latent curve models: A structural equation approach.* Hoboken, NJ: Wiley; 2006.
10. Pinheiro JC, Bates DM: *Mixed-effects models in S and S-plus.* New York: Springer; 2000.
11. Christensen, B. C., Houseman, E. A., Marsit, C. J., Zheng, S., Wrensch, M. R., Wiemels, J. L. et al. (2009). **Aging and environmental exposures alter tissue-specific DNA methylation dependent upon CpG island context**. *PLoS.Genet*, 5, e1000602.
